# Assessing Satisfaction With Online Courses: Spanish Version of the Learner Satisfaction Survey

**DOI:** 10.3389/fpsyg.2022.875929

**Published:** 2022-04-27

**Authors:** Macarena Torrado, María J. Blanca

**Affiliations:** Department of Psychobiology and Methodology of Behavioral Sciences, University of Malaga, Malaga, Spain

**Keywords:** online education, learner satisfaction, reliability analysis, validity evidence, personality traits, motivation

## Abstract

The aim of this study was to develop a Spanish version of the Learner Satisfaction Survey (LSS-S) and to analyze its psychometric properties. The questionnaire was administered to a sample of 1,194 university students. Validity evidence based on the instrument’s internal structure and on relationships with other variables (personality and motivation) were analyzed. In addition, reliability of test scores and differences by gender and area of knowledge were examined. The results revealed a factor structure with adequate fit indices based on five first-order factors (learner–content, learner–instructor, learner–learner, and learner–technology interactions, and general satisfaction) and one second-order factor (total score for academic satisfaction). Scores on the LSS-S were positively correlated with scores on conscientiousness, intrinsic motivation, and identified regulation, and negatively correlated with scores on neuroticism and amotivation. Although the magnitude of correlations with personality traits was small, those with motivational factors were moderate or strong. Reliability of LSS-S factor scores may be considered satisfactory, with McDonald’s omega ranging from 0.80 to 0.86. These results indicate that the LSS-S has satisfactory psychometric properties and that it is an adequate tool for measuring satisfaction with online courses among Spanish learners in higher education.

## Introduction

Although teaching in Spanish universities, as in many other countries around the world, is offered primarily through traditional face-to-face classes, these had to be suspended during 2019 and 2020 due to the COVID-19 pandemic. As a result, faculty had to rely on educational technology so as to adapt their teaching to an online or blended format (Cahapay, 2020; Chung et al., 2022). This has led to increasing interest in the assessment of learners’ satisfaction with this new approach to education, it being recognized that satisfaction is crucial to successful and effective learning (Thurmond et al., 2002; Basith et al., 2020; Barrutia et al., 2021; Flores et al., 2021). Academic satisfaction is generally defined as learners’ appraisal of the extent to which their expectations, needs, and demands have been met during their educational experience (Gento and Vivas, 2003; Moore and Shelton, 2014; González-Peiteado et al., 2017). Given that satisfaction is positively related to learners’ achievement, it is an important factor to consider when designing courses (Bown, 2006; Bernard et al., 2009; González-Peiteado et al., 2017).

Moore (1997) referred to distance education as a concept describing the universe of instructor–learner relationships that exist when learners and instructors are separated by space and/or by time. Moore (1989) described three types of interactions which are essential for effective learning in this context: learner–content interaction, learner–instructor interaction, and learner–learner interaction. The first refers to a learner’s interaction with course contents, lessons, learning activities, learning objects, videos, websites, and projects. The learner-instructor interaction implies two-way communication between learner and instructor, which is necessary to clarify contents, receive and give feedback, and minimize the impact of online education on communication. Finally, learner–learner interaction refers to two-way communication between a learner and other learners. This type of interaction can occur, for example, *via* email and discussion boards. Palloff and Pratt (2001) added a fourth type of interaction that Strachota (2003) labeled learner–technology interaction, referring to learners’ ability and level of comfort in their interactions with online environments (e.g., use of computers, software, and the Internet). Empirical research has shown that these different types of interaction play an important role with regard to achievement outcomes, the experience of meaningful learning, and learner satisfaction (Driver, 2002; Frey and Alman, 2003; Strachota, 2003; Finlay et al., 2004; Chang and Smith, 2008; Bernard et al., 2009; Abrami et al., 2011; Chang, 2013; Kuo et al., 2013; Alqurashi, 2019; Basith et al., 2020; Ngo et al., 2021).

Drawing on the aforementioned interactions, Strachota (2003) developed the Online Satisfaction Survey (OSS), a 42-item self-report instrument that measures learner satisfaction with online courses. In addition to considering the four types of interaction mentioned above (learner–content, learner–instructor, learner–learner, and learner–technology), the OSS also includes a general satisfaction section. Strachota (2003) performed an exploratory factor analysis that provided support for a structure based on these five factors. The reliability of test scores was satisfactory, with Cronbach’s alpha coefficients ranging from 0.89 to 0.97. Strachota also analyzed differences by sociodemographic characteristics of her sample. Overall, the results showed that females were more satisfied than males with learner–instructor interaction. In addition, learners enrolled in health-related studies and liberal arts and sciences courses were significantly more satisfied with learner–content interaction. Learners from the health occupations were also significantly more satisfied with learner–instructor interaction. Business learners were significantly more satisfied with learner–technology interaction than were their peers in the liberal arts and sciences, and those in health occupations. In general, learners in health occupations and the liberal arts and sciences were the most satisfied with online courses.

More recently, Chang (2013) developed a short version of the OSS, which he called the Learner Satisfaction Survey (LSS). The LSS comprises 25 self-report items reflecting the aforementioned five factors, although with some of the questions reworded so that the questionnaire could be used to assess learner satisfaction with different learning settings (i.e., online, blended, or traditional education). Overall, Chang (2013) found that distance learners were less satisfied with their interactions with content, instructors, and other learners than were traditional learners, but more satisfied with technology interactions. In addition, he found no significant difference in satisfaction between male and female students in the online setting. Importantly, however, he did not analyze the psychometric properties of the LSS. Thus, to the best of our knowledge, there is no validity evidence based on the internal structure of the LSS or on relationships between LSS scores and those on other variables.

Using different instruments for measuring academic satisfaction, including *ad hoc* questionnaires, other researchers have found that learner satisfaction with online courses is related to learners’ personality characteristics and motivation. Bower et al. (2001) found that learners who were thinkers, emotionally stable, conscientious, and self-assured (using the 16 Personality Factor Questionnaire) were more likely to be satisfied with online courses. Cohen and Baruth (2017) found that learners who scored higher on openness to experiences and conscientiousness (using the Big Five questionnaire) tended to be more satisfied with online courses. Shih et al. (2013) found that extraversion and conscientiousness (using the NEO Five-Factor Inventory) predicted satisfaction with online courses, although motivation was a stronger predictor of satisfaction than was personality. Maqbool et al. (2020) also found positive correlations between learner satisfaction and learner intrinsic motivation, identified regulation, and external regulation (using the Situational Motivation Scale).

Although there are several instruments in Spanish for measuring learner satisfaction, most of them are focused on face-to-face courses, for example, the Student Satisfaction with University Education scale (Mejías and Martínez, 2009) or the Satisfaction Questionnaire for University Students (Gento and Vivas, 2003). In the online setting, the majority of studies has either used *ad hoc* questionnaires without reporting their psychometric properties (Recio and Cabero, 2005; Bautista et al., 2020; Mercado-Rey et al., 2021) or is focused on a specific approach to or element of online courses (e.g., the use of podcasts or massive, open online courses; Alarcón et al., 2017; Gil-Jaurena et al., 2017; Alarcón and Blanca, 2020). Consequently, there are very few instruments with adequate psychometric properties for measuring satisfaction with online courses among Spanish learners. One exception is the adaptation by González-Peiteado et al. (2017) of the Student Experience Questionnaire for Graduates (Kember and Leung, 2005), which they used to analyze satisfaction with university life of learners who studied at Spain’s National Distance Learning University (the UNED). The adapted version assesses capabilities and the teaching and learning environment through four factors: capabilities, teaching, content, and communication. To our knowledge, however, there are no instruments available in Spanish for measuring learners’ satisfaction with online courses based on the aforementioned four types of interactions.

Given that learner satisfaction plays an important role with regard to academic achievement and is key to successful and effective learning, it is crucial to have adequate tools in our cultural context for measuring this construct in an online environment. The aim of this study was therefore to adapt the LSS into Spanish and to provide evidence of its psychometric properties. The LSS allows a complete evaluation of learner satisfaction in relation to learner–content, learner–instructor, learner–learner, and learner–technology interactions, as well as providing a measure of general satisfaction. This assessment can provide instructors with useful information about the areas most in need of improvement. The main strengths of the LSS are that it can be used at all levels of higher education, its relatively small number of items and its potential applicability to other learning settings (e.g., blended and traditional), thus enabling comparison of learner satisfaction across these different approaches. In adapting the instrument, the Standards for Educational and Psychological Testing (American Educational Research Association et al., 2014) and the recommendations of International Test Commission (2017) were followed. First, confirmatory factor analysis (CFA) was performed to provide validity evidence based on the instrument’s internal structure, including factorial invariance across gender. Second, the reliability of test scores and validity evidence based on relationships between LSS-S scores and scores on measures of personality characteristics and motivation were analyzed. Finally, differences by gender and area of knowledge were analyzed.

## Materials and Methods

### Participants

The sample consisted of 1,194 students (514 males and 680 females) from the University of Malaga (Spain), with a mean age of 21.65 years (*SD* = 5.18). Sample characteristics are shown in Table 1. In the 2020/2021 academic year, the University of Malaga offered 73 undergraduate degrees, 71 Master’s degrees, and 22 doctoral programs, with an approximate enrolment of 36,000 students, of whom 54.55% were women and 45.45% men.

**Table 1:** Sociodemographic characteristics of the sample.

	*N*	%
**Gender**		
Female	680	57
Male	514	43
**Age**		
18–22	924	77.4
23–27	203	17.0
Older than 27 years	67	5.6
**Education**		
Bachelor’s student	1,133	94.9
Master’s student	59	4.9
PhD student	2	0.2
**Area of knowledge**		
Arts and Humanities	202	16.9
Social and Legal Sciences	393	32.9
Health Sciences	156	13.1
Sciences	120	10.0
Engineering and Architecture	323	27.1
**Marital status**		
Single	1,164	97.5
Married	26	2.2
Divorced	3	0.3
Widowed	1	<0.1
**Student status**		
Full-time	1,130	94.6
Part-time	64	5.4
**Work status**		
Full-time	43	3.6
Part-time	165	13.8
Does not work	986	82.6
**Frequency of internet use**		
Less than 5 h a week	6	0.5
5–10 h a week	151	12.6
11–20 h a week	258	21.6
More than 20 h a week	779	65.2

### Instruments

#### Learner Satisfaction Survey

Learner satisfaction survey (LSS; Chang, 2013) is a short version of the Online Satisfaction Survey (Strachota, 2003) and it can be used to assess learner satisfaction with online courses. It comprises 25 self-report items that measure four aspects of interaction (learner–content, learner–instructor, learner–learner, and learner–technology interactions), as well as general satisfaction. Each item is rated on a 4-point Likert scale (1 = *strongly disagree*, 2 = *disagree*, 3 = *agree*, and 4 = *strongly agree*), and higher scores are indicative of higher levels of satisfaction. The instrument was translated into Spanish (hereinafter, the LSS-S) using a back translation method in accordance with the recommendations of the International Test Commission (2017). The Spanish version is available as Supplementary Material of this article.

#### The Situational Motivation Scale

The Situational Motivation Scale (SIMS; Guay et al., 2000), in its Spanish version (Martín-Albo et al., 2009). The SIMS is a self-report inventory of 16 items, each rated on a 7-point Likert scale anchored by 1 (*does not correspond at all*) and 7 (*corresponds exactly*). This instrument measures the level of self-determined motivation in a specific situation (from less to more self-determined), and it comprises four subscales: intrinsic motivation, external regulation, identified regulation and amotivation. Intrinsic motivation refers to performing a behavior only for the pleasure and satisfaction derived from doing it. External regulation refers to performing a behavior to obtain reward or to avoid punishment. Identified regulation is the somewhat internal motivation based on conscious values that are personally important. Finally, amotivation occurs when individuals do not perceive the contingencies between behavior and its consequences; in this case, the behavior is neither extrinsically nor intrinsically motivated, and individuals may feel incompetent. Higher scores are indicative of a higher level of the respective factor. Cronbach’s alpha coefficients in the present sample were 0.89, 0.86, 0.79, and 0.85 for intrinsic motivation, identified regulation, external regulation, and amotivation, respectively.

#### The International Personality Item Pool–Big Five Markers-20

The International Personality Item Pool–Big Five Markers-20 (IPIP-BFM-20; Goldberg, 1999; Donnellan et al., 2006), in its Spanish version (Martínez-Molina and Arias, 2018). This self-report test comprises 20 items that are rated on a 5-point Likert scale anchored by 1 (*very inaccurate as a description of you*) and 5 (*very accurate as a description*). The instrument comprises five factors: extraversion, agreeableness, conscientiousness, neuroticism, and openness. People who score higher may be described as: active, assertive, and talkative (extraversion); trustful, kind, and helpful (agreeableness); organized, diligent, and efficient (conscientiousness); anxious, nervous, prone to anger, and irritation (neuroticism); and cognitively open, creative, and introspective (openness). Those who score lower may be described as: introverted, reserved, and quiet (extraversion); distrustful, selfish, and rude person (agreeableness); unsystematic, unconcerned with order and planning, and negligent (conscientiousness); relaxed, calm, and imperturbable (neuroticism); and unintellectual, unimaginative, and unreflective (openness). Cronbach’s alpha coefficients in the present sample were 0.79, 0.73, 0.72, 0.68, and 0.60 for extraversion, agreeableness, conscientiousness, neuroticism, and openness, respectively. The last two alpha values are below the usually recommended cut-off of 0.70. However, given that the factors comprise only four items and that the IPIP-BFM-20 is one of the most widely used short scales based on the Big Five model (Martínez-Molina and Arias, 2018), we considered these values to be acceptable.

### Procedure

The study procedures were carried out in accordance with the Declaration of Helsinki and were approved by the Experimentation Ethics Committee of the University of Malaga (45-2021-H). A questionnaire comprising the three aforementioned instruments was made available through the website of the University of Malaga for 15 days at the end of the 2020–21 academic year. All participants were informed about the objective of the study, and it was made clear that their responses would remain anonymous. They all provided informed consent prior to answering the online questionnaire, which took around 10 min to complete. There were no missing data because the online questionnaire could not be submitted unless all the questions had been answered.

### Data Analysis

A descriptive analysis of LSS-S items was performed, using IBM SPSS 25 to compute means, standard deviations, and skewness and kurtosis coefficients.

Next, and with the aim of obtaining validity evidence based on the internal structure of the LSS-S, a CFA was performed using the EQS 6.4 program (Bentler, 2006). Given the dimensions of the questionnaire, a structure based on five first-order factors and one second-order factor was tested. Configural and metric invariance were also analyzed to establish whether the number of factors and factor-loading patterns was the same across genders. Invariance was tested by fitting a series of nested CFA models with increasing constraints, following the procedure suggested by Byrne and Stewart (2006) and Byrne (2008). It began by determining the baseline model for each group separately and then tested the configural invariance by constraining the factor structure to be equal across genders. Finally, metric invariance was tested by constraining the first-order factor loadings and second-order factor loadings. Invariance was assessed by comparing the comparative fit index (CFI) of the configural model with the CFI of all subsequent invariance models. The equality of constraints was considered to be tenable if the decrease in CFI in the most constrained model was less than or equal to 0.01 in relation to the configural model (Cheung and Rensvold, 2002). All CFA analyses were performed using robust maximum likelihood estimators based on the polychoric correlation matrix of items. The Satorra-Bentler chi-square (S-B *χ*^2^) was computed with the following goodness-of-fit indices: the CFI (Bentler, 1990), the non-normed fit index (NNFI; Bentler and Bonett, 1980), and the root mean square error of approximation (RMSEA; Browne and Cudeck, 1993; Steiger, 2000). Values of the CFI and the NNFI above 0.95 are generally considered a good fit (Hu and Bentler, 1999), whereas values of the RMSEA between 0.06 and 0.08 indicate a reasonable fit (Browne and Cudeck, 1993; MacCallum et al., 1996), and those below 0.06 a good fit (Hu and Bentler, 1999).

In order to examine the reliability of test scores, the internal consistency of LSS-S factor scores was analyzed by computing McDonald’s omega coefficient. Values of 0.70 or higher are generally considered acceptable (Campo-Arias and Oviedo, 2008; Viladrich et al., 2017).

To obtain validity evidence based on relationships with other variables, Pearson correlation coefficients were computed between scores on the LSS-S and scores on the SIMS and IPIP-BFM-20. Values around 0.10 were considered as small correlations, those around 0.30 as moderate, and values of 0.50 or higher as indicating strong correlations (Cohen, 1988).

Finally, mean differences by gender were analyzed using a *t*-test for independent samples and differences by area of knowledge using one-way analysis of variance (ANOVA) with Bonferroni correction for multiple comparisons.

## Results

### Descriptive Analysis

Table 2 shows results from the descriptive analysis of LSS-S item scores. Overall, the mean item scores were around 2 (rated from 1 to 4). The skewness and kurtosis indices indicated some deviation from the normal distribution. Bentler (2006) pointed out that kurtosis is the key issue for covariance structure analysis and recommended using the robust estimation method when data are not normal. He also suggested that values of Mardia’s normalized estimate higher than 5 or 6 may be indicative of data that are non-normally distributed. The value of this coefficient in our data was 49.49, justifying the use of the robust method to perform the CFA.

**Table 2:** Means (M), standard deviation (SD), skewness, and kurtosis for items of the LSS-S (*N* = 1,194).

Items	*M*	*SD*	Skewness	Kurtosis
**Learner–content interaction**				
The course notes, lessons, or lecture used in this course have facilitated my learning	2.41	0.97	0.07	−1.00
The assignments or projects in this course have facilitated my learning	2.35	0.96	0.08	−0.98
Preparation for quiz/exams in this course has facilitated my learning	2.02	1	0.61	−0.76
The learning activities in this course have required application of problem solving skills which facilitated my learning	2.32	0.96	0.17	−0.96
The learning activities in this course have required critical thinking which facilitated my learning	2.28	1.02	0.17	−1.13
**Learner–instructor interaction**				
In this course the teachers have been active members of discussion groups, offering direction to our discussions	2.10	0.99	0.41	−0.98
I have received timely feedback from my teachers	2.16	0.98	0.30	−1.01
I have been able to get individualized attention from my teachers when needed	2.47	1.08	−0.05	−1.29
In this course the teachers have functioned as the facilitators of the course by continuously encouraging communication	2.21	0.99	0.26	−1.05
When I have attended the course, the teacher knew I was present	2.08	1.07	0.53	−1.03
**Learner–learner interaction**				
In this course the discussion activities have provided opportunity for problem solving with other students	1.98	0.98	0.58	−0.82
This course has created a sense of community among students	1.88	1.01	0.76	−0.70
In this course I have been able to share my viewpoint with other students	2.34	1.06	0.13	−1.23
In this course I have received timely feedback from other students	2.11	1	0.37	−1.06
In this course I have been encouraged to discuss ideas and concepts covered with other students	1.96	1	0.62	−0.89
**Learner–technology interaction**				
I enjoy working with computers	2.56	1.11	−0.12	−1.34
Computers make me much more productive	2.39	1.12	0.11	−1.36
I am very confident in my abilities to use computers	2.85	1.06	−0.48	−1.02
Some computer software packages definitely make learning easier	2.93	1.01	−0.63	−0.71
Computers are good aids to learning	3.04	0.93	−0.73	−0.31
**General satisfaction**				
I am very satisfied with this course	1.93	0.96	0.67	−0.64
I would like to take other courses with the same learning setting	2.24	1.08	0.31	−1.22
This course definitely meets my learning needs	1.84	0.93	0.85	−0.28
I would definitely recommend this course to others	1.97	0.96	0.62	−0.69
I feel this course is as effective as other courses with different learning settings (for example, traditional face-to-face learning)	1.77	1.05	1.10	−0.26

### Validity Evidence Based on Internal Structure

The structure based on five first-order factors and one second-order factor showed a good model fit for the total sample (Table 3). Values of the CFI and NNFI were above 0.95, while the RMSEA was lower than 0.06.

**Table 3:** Fit indices for the second-order factor model of the Spanish version of the LSS.

Model	S-B *x*^2^	*df*	CFI	NNFI	RMSEA	**Ʌ**CFI
Total sample	1136.81	270	0.992	0.991	0.052 [0.049, 0.055]	
Male	648.51	270	0.993	0.992	0.052 [0.047, 0.057]	
Female	753.83	270	0.992	0.991	0.051 [0.047, 0.056]	
Configural invariance	1394.95	540	0.992	0.991	0.052 [0.048, 0.055]	
First-order loadings	1428.19	560	0.992	0.992	0.051 [0.048, 0.054]	<0.001
Second-order loadings	1435.76	565	0.992	0.992	0.051 [0.048, 0.054]	<0.001

*N* = 1,194. S-B *x*^2^, Satorra–Bentler; CFI, comparative fit index; NNFI, non-normed fit index; RMSEA, root mean square error of approximation; CI, confidence interval; and Ʌ CFI, CFI configural invariance model—CFI more constrained model.

**Figure 1:**
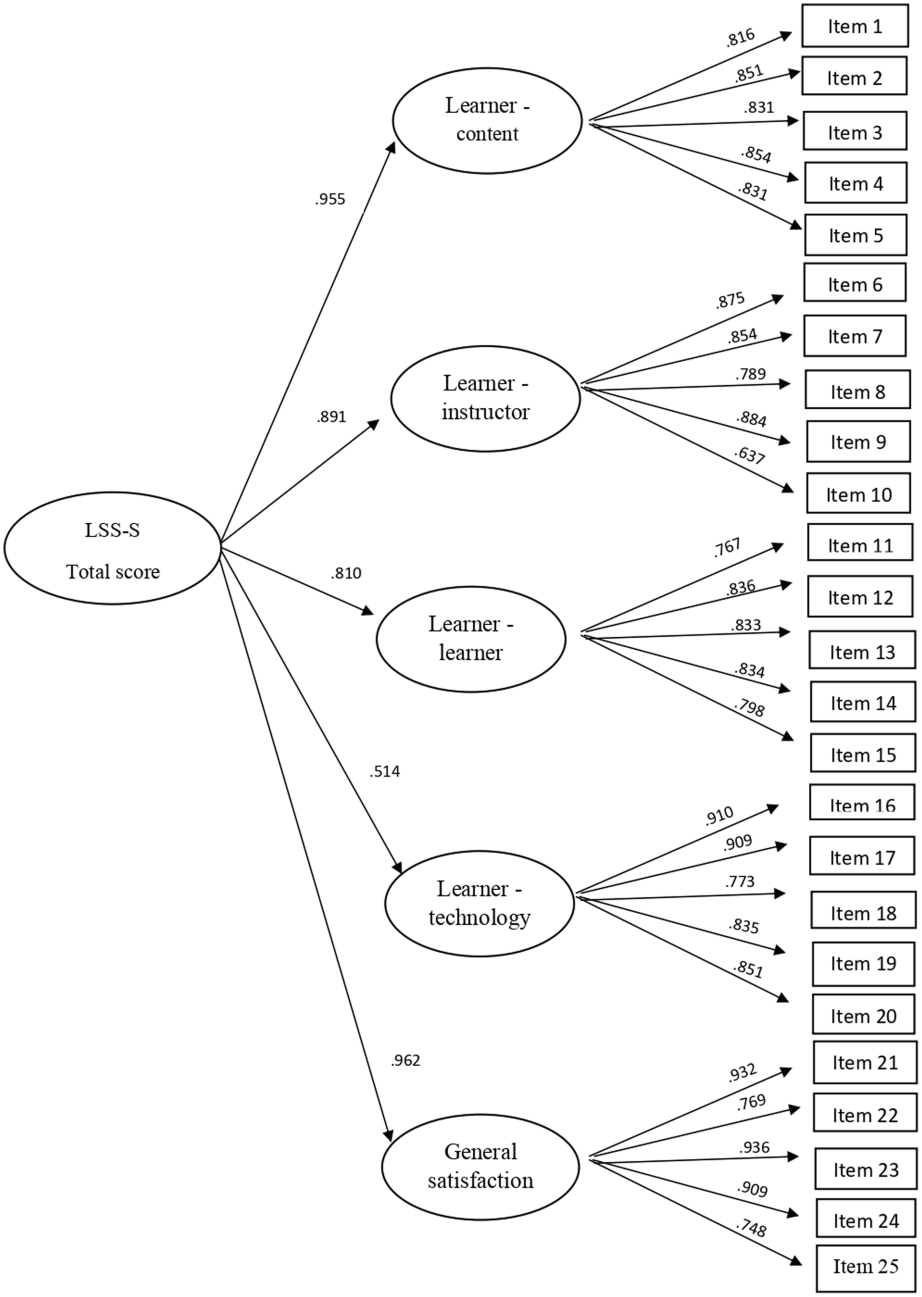
Values of standardized parameters for a factor structure based on five first-order factors and one second-order factor.

Regarding factorial invariance across genders, the goodness-of-fit indices were satisfactory in all models (configural and metric invariance). In addition, the decrease in CFI value from the configural model to the most constrained models did not exceed 0.001. The values of standardized parameters for the total sample are shown in Figure 1. All parameters were statistically significant.

### Reliability of LSS-S Scores

The McDonald’s omega coefficients were 0.83, 0.80, 0.80, 0.85, and 0.86 for learner–content interaction, learner–instructor interaction, learner–learner interaction, learner–technology interaction, and general satisfaction, respectively. For total scores, the omega coefficient was 0.96. This indicates satisfactory reliability of test scores.

### Validity Evidence Based on Relationships With Other Variables

Table 4 shows descriptive statistics for the IPIP-BFM-20 and the SIMS, including the mean, standard deviation, and minimum and maximum values for each subscale.

**Table 4:** Means (M), standard deviation (SD), and minimum (Min) and maximum values (Max) for subscales of the IPIP-BFM-20 and the SIMS.

Variables	*M*	*SD*	Min	Max
**IPIP-BFM-20**				
Extraversion	11.98	3.94	4	20
Agreeableness	15.84	3.12	4	20
Conscientiousness	14.35	3.49	4	20
Neuroticism	12.67	3.52	4	20
Openness	13.76	2.37	4	20
**SIMS**				
Intrinsic motivation	12.60	6.59	4	28
Identified regulation	18.31	6.64	4	28
External regulation	19.10	6.24	4	28
Amotivation	14.56	6.81	4	28

*N* = 1194.

Table 5 shows results from the Pearson correlations between LSS-S scores and scores on the IPIP-BFM-20 and SIMS. Overall, and with regard to the IPIP-BFM-20, positive relationships were observed between LSS-S factor scores and scores on conscientiousness, and negative relationships between learner–technology interaction and total scores and scores on neuroticism. These correlation values were statistically significant, although their magnitudes were small (around 0.10). With respect to the SIMS, LSS-S factor scores were positively and significantly correlated with scores on intrinsic motivation and identified regulation, and negatively correlated with amotivation scores. These correlation values were moderate or strong.

**Table 5:** Pearson correlation coefficients between scores on the LSS-S and scores on the IPIP-BFM-20 and the SIMS.

Variables	Learner–content	Learner–instructor	Learner–learner	Learner–technology	General satisfaction	LSS-S total score
**IPIP-BFM-20**						
Extraversion	<0.01	0.02	0.08^*^	−0.05	<0.01	0.01
Agreeableness	0.10^***^	0.07^*^	0.09^**^	−0.01	0.07^*^	0.09^*^
Conscientiousness	0.12^***^	0.12^***^	0.09^**^	0.10^***^	0.16^***^	0.15^*^
Neuroticism	−0.07^*^	−0.06^*^	−0.08^**^	−0.14^***^	−0.08^**^	−0.15^*^
Openness	−0.02	<−0.01	−0.07	0.06	<−0.01	<0.01
**SIMS**						
Intrinsic motivation	0.58^***^	0.50^***^	0.50^***^	0.32^***^	0.62	0.61^*^
Identified regulation	0.44^***^	0.37^***^	0.37^***^	0.29^***^	0.44^***^	0.47^*^
External regulation	0.01	<0.01	−0.02	0.07^*^	−0.04	<0.01
Amotivation	−0.39^***^	−0.37^***^	−0.30^***^	−0.18^***^	−0.42^***^	−0.40^*^

*N* = 1194.

*** *p < 0.001*;

** *p < 0.01*;

* *p < 0.05*.

### Differences by Gender and Area of Knowledge

Considering the total sample, the mean of factor scores ranged from 9.76 (general satisfaction) to 13.77 (learner–technology interaction), with a mean for total score on the LSS-S of 56.20 (out of a maximum of 100). This total score is calculated by summing scores on the 25 items.

Table 6 shows the results from the *t*-test for independent samples. Statistically significant differences between female and male students were found for all LSS-S factor scores. Females scored higher on all factors, except for the learner–technology factor, on which scores were higher among male students.

**Table 6:** Value of the t-test for independent samples and probability (bilateral) for total sample and by gender.

Factors	Total sample	*M* (*SD*)		*t*	*p*
		Female	Male		
Learner–content	11.37 (4.10)	11.85 (4.03)	10.74 (4.10)	4.68	<0.001
Learner–instructor	11.03 (4.14)	11.49 (4.09)	10.41 (4.11)	4.49	<0.001
Learner–learner	10.28 (4.08)	10.63 (3.99)	9.82 (4.16)	3.41	0.001
Learner–technology	13.77 (4.42)	13.40 (4.26)	14.25 (4.59)	−3.28	0.001
General satisfaction	9.76 (4.21)	10.10 (4.14)	9.30 (4.26)	3.29	0.001
LSS-S total score	56.20 (17.08)	57.48 (16.78)	54.52 (17.35)	2.97	0.003

Female (*n* = 680); Male (*n* = 514).

Table 7 shows the results from the one-way ANOVA by area of knowledge. The results showed statistically significant differences on all LSS-S factors. Overall, students from the field of Engineering and Architecture obtained the lowest scores for satisfaction with online courses, with the exception of the learner–technology factor, on which they had the highest mean score.

**Table 7:** Means (*M*), standard deviation (*SD*) in parentheses, *F* statistics, probability, and mean comparisons by area of knowledge.

Variables	1	2	3	4	5	*F*	*p*	Comparisons (Bonferroni)
Learner–content	11.25 (4.00)	11.83 (4.29)	12.04 (4.17)	11.13 (4.09)	10.66 (3.77)	4.94	0.001	2 vs. 5, 3 vs. 5
Learner– instructor	11.53 (4.22)	11.42 (4.17)	12.04 (4.28)	10.23 (3.90)	10.03 (3.81)	10.07	<0.001	1 vs. 5, 2 vs. 5, 3 vs. 5, 3 vs. 4
Learner–learner	10.28 (4.03)	10.67 (4.28)	11.18 (4.01)	10.35 (4.16)	9.34 (3.69)	7.19	<0.001	2 vs. 5, 3 vs. 5
Learner–technology	12.59 (4.57)	13.89 (4.26)	13.26 (4.08)	13.28 (4.48)	14.78 (4.43)	9.02	<0.001	1 vs. 5, 3 vs. 5, 4 vs. 5, 1 vs. 2
General satisfaction	9.73 (4.19)	10.32 (4.33)	10.39 (4.38)	9.65 (4.13)	8.83 (3.85)	6.75	<0.001	2 vs. 5, 3 vs. 5
LSS-S Total score	55.38 (17.30)	58.13 (17.94)	58.90 (17.24)	54.65 (17.27)	53.64 (15.28)	4.46	0.001	2 vs. 5, 3 vs. 5

1, Arts and Humanities (*n* = 202); 2, Social and Legal Sciences (*n* = 393); 3, Health Sciences (*n* = 156); 4, Sciences (*n* = 120); and 5, Engineering and Architecture (*n* = 323).

## Discussion

The aim of this study was to analyze the psychometric properties of the LSS-S in a sample of Spanish higher education learners. Validity evidence based on the internal structure was obtained, as well as based on relationships with other variables (personality and motivation). In addition, the reliability of test scores and differences by gender and area of knowledge were examined. Overall, the results indicate that the LSS-S has satisfactory psychometric properties and that it is an adequate tool for measuring satisfaction with online courses.

Regarding validity evidence based on the internal structure, the results obtained by CFA indicated a second-order structure with good fit indices. The first-order factors (i.e., learner–content, learner–instructor, learner–learner, and learner–technology interactions, and general satisfaction) are consistent with those described in previous studies (Strachota, 2003; Chang, 2013). The second-order factor subsumes the scores obtained on these factors and supports the use of a total score for satisfaction with online courses. In addition, factorial invariance was found across genders, showing configural and metric invariance. This indicates that the LSS-S has a stable factor structure across male and female students. The reliability of the LSS-S factor scores was also satisfactory, with values of McDonald’s omega ranging from 0.80 to 0.86, similar to the Cronbach’s alpha coefficients obtained with the original version of the instrument (Strachota, 2003).

In terms of validity evidence based on relationships with other variables, correlations of small magnitude were found between LSS-S factor scores and scores on the IPIP-BFM-20. Overall, the most relevant finding was the positive correlations between LSS-S factor scores and scores on conscientiousness, as well as the negative correlation between both learner–technology interaction and total scores with neuroticism. These results indicate that people who are organized, diligent, and efficient tend to be more satisfied with online courses and with learner–content, learner–learner, learner–instructor, and learner–technology interactions. Conversely, people who are anxious, nervous, and prone to anger and irritation tend to be less satisfied with technology and, accordingly, with online learning. These findings are partially consistent with other research that has likewise reported a positive correlation between conscientiousness and academic satisfaction with online courses (Shih et al., 2013; Cohen and Baruth, 2017). The results are also in line with those of Bower et al. (2001), who found that those learners who were emotionally stable and conscientious were more likely to be satisfied with online courses.

The present analysis also showed that LSS-S factor scores correlated positively with intrinsic motivation and identified regulation, and negatively with amotivation, although in this case the magnitude of relationships was moderate or strong. These results suggest that learners who enroll in an online course for the pleasure and satisfaction derived from doing it, and whose conscious values provide them with internal motivation to achieve the course objectives tend to be more satisfied with online courses and with the different types of interactions that form part of the teaching-learning process. Conversely, learners who do not perceive the contingencies between behavior and its consequences tend to be generally less satisfied with the online format. These findings are consistent with previous research suggesting that academic satisfaction is more strongly related to motivation than to personality characteristics (Shih et al., 2013).

Regarding gender differences, the results showed that females reported a higher level of academic satisfaction, except in relation to learner–technology interactions, where scores were higher among male students. Strachota (2003) only found that females were more satisfied than males with learner–instructor interaction, whereas Chang (2013) did not find significant differences between male and female students in satisfaction with online courses. These discrepancies may be related to procedural issues. For example, in the studies by Strachota (2003) and Chang (2013), learners voluntarily chose the online course, and there was a higher proportion of female and full-time workers in the sample. Conversely, in the present study, learners were obliged to take part in online education (due to the COVID-19 pandemic), the sample consisted of a similar number of males and females, and the majority of participants was full-time students and did not work.

With respect to differences by area of knowledge, students from the field of Engineering and Architecture had the lowest scores for academic satisfaction with online courses, except in relation to learner–technology interactions, where they scored the highest. This finding may be related to the contents of these degree programs, which are more technical and require the use of different educational resources to those used in other areas of knowledge, as a result of which, these students found it more challenging to adapt to exclusively online learning. The fact that students from Engineering and Architecture were more satisfied with learner–technology interactions may be a reflection of their greater understanding of technology and a greater ability to manage it.

Overall, the LSS-S scores obtained in the present sample indicate that learners were moderately satisfied with online courses. The mean for total scores on the LSS-S was 56.20, out of a maximum of 100, which indicates that the teaching-learning process needs to be improved. Scores on the LSS-S have practical implications, as examining them can provide instructors with useful information about the areas most in need of improvement. In the present sample, for instance, scores suggest that particular attention would need to be paid to learner–learner interactions. The fact that improvements are indicated is not, however, surprising, insofar as the COVID-19 pandemic meant that faculty had to adapt all their face-to-face teaching to an online format in a short period of time, including revising contents and implementing the use of new resources and technology. When adapting their teaching to an online environment, instructors must take into account the different types of interaction involved and remain mindful that teaching in this environment requires specific skills and techniques to create an effective learning experience. Course design is crucial for promoting learning, and it is important to ensure the inclusion of meaningful instructional content and activities that increase students’ motivation and facilitate their reasoning, problem solving, and critical thinking skills. Instructors should also be proactive in promoting and facilitating online communication with their students, providing feedback, and supporting them with individualized attention. Similarly, they should foster the exchange of information among learners, encouraging engagement with peers through the discussion of ideas, opinions, etc. Finally, students must also have the technological skills required for participation in online learning environments. In this respect, universities must be proactive, providing technological support and designing activities that help students to improve their skills and succeed in online learning.

This study has a number of limitations that should be acknowledged. First, participants were recruited solely through the University of Malaga and this might limit the generalizability of results. It would thus be interesting in future studies to include learners from other geographic areas of Spain, as well as from Latin American countries. Second, validity evidence for the LSS-S was provided based on associations with motivation and personality traits, and it would be interesting in future research to study the relationship with other variables such as self-efficacy, stress, and academic achievement. Finally, relationships between variables were examined using a correlational analysis, and hence, no causality can be inferred. Further longitudinal studies are therefore needed to extend knowledge about the nature of these relationships.

Despite these limitations, this study has a number of strengths, not least that it is the first study to analyze the psychometric properties of the LSS in a relatively large sample of university learners. Specifically, validity evidence based on the instrument’s internal structure have been provided, including factorial invariance across genders, as well as validity evidence based on relationships with other variables such as personality characteristics and motivation (variables that have not previously been considered in relation to the LSS). Overall, the results indicate that the LSS-S has satisfactory psychometric properties and that it is an adequate tool for measuring satisfaction with online courses among Spanish learners in higher education. Importantly, the LSS-S can also be used to assess academic satisfaction with traditional and blended learning approaches. Further research is required to provide evidence of its psychometric properties in these contexts.

## Conclusion

The present study provides evidence of the psychometric properties of the LSS-S in a sample of Spanish university learners, a population not previously studied. The results reveal a factor structure with adequate fit indices based on five first-order factors (learner–content, learner–instructor, learner–learner, and learner–technology interactions, and general satisfaction) and one second-order factor (total score of satisfaction). Reliability of LSS-S factor scores may be considered satisfactory. Scores on the LSS-S were positively correlated with scores on conscientiousness, intrinsic motivation, and identified regulation, and negatively correlated with scores on neuroticism and amotivation. Although the magnitude of correlations with personality traits was small, those with motivational factors were moderate or strong. The results indicate that the LSS-S has satisfactory psychometric properties and that it is an adequate tool for measuring satisfaction with online courses among Spanish learners in higher education. Furthermore, it is a short and simple questionnaire that can be administered collectively. The scores obtained provide instructors with direct feedback from learners regarding their level of satisfaction with different aspects of the teaching-learning process, thereby highlighting areas in need of improvement. Learner satisfaction is a key factor for successful and effective learning and instructors should take into account the different types of interaction involved to create an effective learning environment.

## Data Availability Statement

The original contributions presented in the study are included in the article/Supplementary Material, and further inquiries can be directed to the corresponding author.

## Ethics Statement

The studies involving human participants were reviewed and approved by the Experimentation Ethics Committee of the University of Malaga (45-2021-H). The patients/participants provided their written informed consent to participate in this study.

## Author Contributions

All authors listed have made a substantial, direct, and intellectual contribution to the work and approved it for publication.

## Conflict of Interest

The authors declare that the research was conducted in the absence of any commercial or financial relationships that could be construed as a potential conflict of interest.

## Publisher’s Note

All claims expressed in this article are solely those of the authors and do not necessarily represent those of their affiliated organizations, or those of the publisher, the editors and the reviewers. Any product that may be evaluated in this article, or claim that may be made by its manufacturer, is not guaranteed or endorsed by the publisher.
