# Supplementary material for: Assessing Satisfaction With Online Courses: Spanish Version of the Learner Satisfaction Survey
Source: Front Psychol. 2022 Apr 27;13:875929. doi: 10.3389/fpsyg.2022.875929 (PMC9094622; doi:10.3389/fpsyg.2022.875929)
Supplement: Supplementary file 1 [file Table_1.DOCX]

**CUESTIONARIO DE SATISFACCIÓN DEL ESTUDIANTE CON LA EDUCACIÓN ONLINE (LSS-S)**

Nos interesa conocer tu experiencia personal en este curso. Por favor, indica en qué medida estás de acuerdo o en desacuerdo con cada una de las siguientes afirmaciones, marcando con una **X** la opción que más se ajuste a tu opinión: totalmente en desacuerdo, en desacuerdo, de acuerdo, totalmente de acuerdo. Por favor, contesta con sinceridad.

|  | Totalmente en desacuerdo | En desacuerdo | De acuerdo | Totalmente de acuerdo |
| --- | --- | --- | --- | --- |
| 1. Los apuntes, temario o clases empleados en las asignaturas han facilitado mi aprendizaje | ◻ | ◻ | ◻ | ◻ |
| 2. Las tareas y proyectos realizados en las asignaturas han facilitado mi aprendizaje | ◻ | ◻ | ◻ | ◻ |
| 3. La preparación para los tests/exámenes en las asignaturas han facilitado mi aprendizaje | ◻ | ◻ | ◻ | ◻ |
| 4. He tenido que usar habilidades para la resolución de tareas en las actividades de aprendizaje de las asignaturas, lo que ha facilitado mi aprendizaje | ◻ | ◻ | ◻ | ◻ |
| 5. Las actividades realizadas en las asignaturas han requerido que use un pensamiento crítico que ha facilitado mi aprendizaje | ◻ | ◻ | ◻ | ◻ |
| 6. El profesorado de las asignaturas ha sido parte activa de los grupos de discusión y nos ha guiado en las discusiones | ◻ | ◻ | ◻ | ◻ |
| 7. El profesorado nos ha enviado comentarios y observaciones oportunas | ◻ | ◻ | ◻ | ◻ |
| 8. He recibido atención individualizada por parte de mis profesores/as cuando la he necesitado | ◻ | ◻ | ◻ | ◻ |
| 9. El profesorado de las asignaturas ha actuado como moderador y ha fomentado la comunicación | ◻ | ◻ | ◻ | ◻ |
| 10. Cuando he asistido a las clases, los/as profesores/as sabían que estaba presente | ◻ | ◻ | ◻ | ◻ |
| 11. Las actividades de discusión de las asignaturas han supuesto una oportunidad para resolver problemas con otros/as estudiantes | ◻ | ◻ | ◻ | ◻ |
| 12. Las asignaturas han creado un sentimiento de comunidad entre los/as estudiantes | ◻ | ◻ | ◻ | ◻ |
| 13. He podido compartir mi punto de vista con otros/as estudiantes en las asignaturas | ◻ | ◻ | ◻ | ◻ |
| 14. En las asignaturas he recibido comentarios oportunos de otros/as estudiantes | ◻ | ◻ | ◻ | ◻ |
| 15. Me he sentido alentado a discutir con otros/as estudiantes sobre las ideas y conceptos presentados en las asignaturas | ◻ | ◻ | ◻ | ◻ |
| 16. Disfruto trabajando con ordenadores | ◻ | ◻ | ◻ | ◻ |
| 17. Los ordenadores me hacen más productivo | ◻ | ◻ | ◻ | ◻ |
| 18. Me siento muy seguro/a de mis habilidades con los ordenadores | ◻ | ◻ | ◻ | ◻ |
| 19. Estoy seguro/a de que algunos programas de ordenador facilitan el aprendizaje | ◻ | ◻ | ◻ | ◻ |
| 20. Los ordenadores son una buena ayuda para el aprendizaje | ◻ | ◻ | ◻ | ◻ |
| 21. Me siento muy satisfecho/a con las asignaturas | ◻ | ◻ | ◻ | ◻ |
| 22. Me gustaría que en otras asignaturas se usaran las mismas técnicas de aprendizaje | ◻ | ◻ | ◻ | ◻ |
| 23. Las asignaturas se adaptan totalmente a mis necesidades de aprendizaje | ◻ | ◻ | ◻ | ◻ |
| 24. Definitivamente recomendaría las asignaturas a otras personas | ◻ | ◻ | ◻ | ◻ |
| 25. Siento que las asignaturas son igual de efectivas que otras con diferentes metodologías de aprendizaje (por ejemplo, con las clases presenciales) | ◻ | ◻ | ◻ | ◻ |
